# Cancer and Radiosensitivity Syndromes: Is Impaired Nuclear ATM Kinase Activity the Primum Movens?

**DOI:** 10.3390/cancers14246141

**Published:** 2022-12-13

**Authors:** Laura El Nachef, Elise Berthel, Mélanie L. Ferlazzo, Eymeric Le Reun, Joelle Al-Choboq, Juliette Restier-Verlet, Adeline Granzotto, Laurène Sonzogni, Michel Bourguignon, Nicolas Foray

**Affiliations:** 1 Inserm, U1296 Unit, Radiation: Defense, Health and Environment, Centre Léon-Bérard, 69008 Lyon, France; 2 Department of Biophysics and Nuclear Medicine, Université Paris Saclay (UVSQ), 78035 Versailles, France

**Keywords:** cancer, genetic syndromes, radiosensitivity, ATM protein

## Abstract

**Simple Summary:**

A review of the molecular and cellular features of the major cancer syndromes associated with radiosensitivity revealed the importance of the ATM protein, either as an impaired kinase in the nucleus or as a complex in the cytoplasm, with the mutated protein responsible for the syndrome.

**Abstract:**

There are a number of genetic syndromes associated with both high cancer risk and clinical radiosensitivity. However, the link between these two notions remains unknown. Particularly, some cancer syndromes are caused by mutations in genes involved in DNA damage signaling and repair. How are the DNA sequence errors propagated and amplified to cause cell transformation? Conversely, some cancer syndromes are caused by mutations in genes involved in cell cycle checkpoint control. How is misrepaired DNA damage produced? Lastly, certain genes, considered as tumor suppressors, are not involved in DNA damage signaling and repair or in cell cycle checkpoint control. The mechanistic model based on radiation-induced nucleoshuttling of the ATM kinase (RIANS), a major actor of the response to ionizing radiation, may help in providing a unified explanation of the link between cancer proneness and radiosensitivity. In the frame of this model, a given protein may ensure its own specific function but may also play additional biological role(s) as an ATM phosphorylation substrate in cytoplasm. It appears that the mutated proteins that cause the major cancer and radiosensitivity syndromes are all ATM phosphorylation substrates, and they generally localize in the cytoplasm when mutated. The relevance of the RIANS model is discussed by considering different categories of the cancer syndromes.

## 1. Introduction

### 1.1. The Syndromes Combining Cancer Proneness and Radiosensitivity

To date, radiotherapy (RT) remains as one of the major tools for cancer treatment to control and eradicate cancer cells. Radiation oncologists have to face a double challenge: kill the tumor and spare the healthy tissues. After more than one century of anti-cancer RT, a considerable amount of data has been accumulated to better understand the molecular mechanisms underlying the cellular response of tumors and healthy tissues to ionizing radiation (IR) but also to some chemotherapy agents that mimic IR [1,2,3,4,5]. Particularly, in 5 to 20% of RT-treated cancer cases, some adverse tissue reactions, ranging from radiation-induced (RI) blushes without consequence to fatal reactions, can occur during or after RT, which oblige clinicians to modify or stop the scheduled treatment [6,7,8]. The prediction and prevention of such adverse reactions of exposed tissue, called radiosensitivity reactions or radiotoxicities, is a big challenge in radiotherapy and in radiobiology [9]. Hence, cancer proneness can be associated with a large spectrum of RI adverse reactions, therefore defining some “syndromes” that link both cancer proneness and radiosensitivity. These particular syndromes raise the question of the interplay between the cellular transformation process leading to cancer and an abnormal response to IR leading to tissue injuries [4,5].

### 1.2. Interplay between DNA Damage Repair and Signaling and Cell Cycle Checkpoint Arrests

While carcinogenesis and the cellular transformation process have been mainly associated with a lack of control of cell cycle checkpoint arrests enabling cellular proliferation, radiosensitivity has been generally linked to dysfunctions in chromosomal and DNA damage signaling and repair pathways [9,10,11]. However, such interplay between cancer and radiosensitivity remains unclear. For example, some mutations of the *LIG4* and *XP* genes that cause cancer and radiosensitivity syndromes have been shown to directly impact DNA damage signaling and repair but not on the cell cycle checkpoint arrest control. Conversely, some mutations of the *p53* or *CHK2* genes that cause some other cancer and radiosensitivity syndromes have been shown to directly impact the cell cycle checkpoint arrest controls but not DNA damage signaling and repair [4,5]. Lastly, the mutations of *NF1* or *TSC* genes, whose biological function directly impacts DNA damage signaling and repair and cell cycle checkpoint arrest controls, have been associated with cancer and radiosensitivity [12,13]. Hence, these examples reveal not only that the molecular bases of cancer proneness remain to be more documented, but also their link with radiosensitivity is still poorly understood.

### 1.3. The ATM Protein as the Crossroads of Cancer and Radiosensitivity

**Figure 1:**
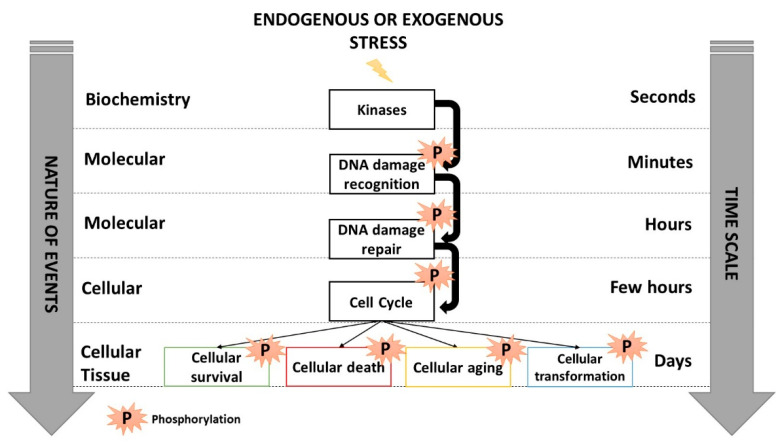
Schematic view of the cascades of phosphorylations occurring in response to IR activated by kinases that trigger DNA damage recognition, repair, cell cycle arrests and cellular death. Inspired from [15].

IR generally causes three major types of DNA damage: base damage (BD), single-strand breaks (SSB) and DNA double-strand breaks (DSB). Throughout the natural selection, cells are equipped with various pathways to manage BD, SSB and DSB specifically [4,14]. Each type of DNA damage triggers a specific ordered succession of enzymatic steps, frequently operating via a cascade of phosphorylations directed by upstream kinases: DNA damage is firstly recognized several minutes post-stress, is repaired for a few hours, cell cycle checkpoint arrests are activated some hours post-stress, and then cellular death is triggered several hours thereafter together with the initiation of cell transformation or aging processes (Figure 1) [15]. Any lack or impairment of the most upstream molecular events occurring immediately after the DNA damage formation results in the most severe biological effects and may condition the next molecular and cellular steps [15]. Such an RI cascade of phosphorylations reveals that RI DNA damage formation, recognition and repair occur systematically before cell cycle checkpoint arrests, suggesting that cells do not arrest their cycle to repair their DNA damage since the great majority of them are already recognized and/or repaired [10,16].

Among the RI DNA damage, DSB appears to be the key damage of cell lethality [17,18]. Among the kinases involved in both DSB signaling and repair and cell cycle checkpoint arrest control, the ATM kinase is considered as a major actor of the response to IR, since it phosphorylates numerous protein substrates belonging to the RI cascade of phosphorylations evoked above [15,19]. More recently, we have documented the role of the RI ATM nucleoshuttling (RIANS) in the individual response to IR and showed that the RIANS may serve as a reliable marker of radiosensitivity, whatever the nature and the dose of IR [8,18,20]. Briefly, after exposure to IR, the cytoplasmic ATM dimers dissociate as ATM monomers and diffuse in the nucleus. Once in the nucleus, ATM monomers phosphorylate the X variant of the H2A histone protein (γH2AX), which triggers the recognition of the RI DSB by the non-homologous end-joining (NHEJ) pathway, the most predominant DSB signaling and repair pathway in humans [8,18,20]. However, the RIANS can be delayed by the overexpression of some cytoplasmic substrates of ATM (called X-proteins). These X-proteins hold SQ and TQ domains that are specifically phosphorylated by ATM after exposure to IR [19]. The X-proteins sequestrate the RI ATM monomers, decrease their flux in the nucleus and therefore affect the nuclear ATM kinase activity and the number of DSB recognized by NHEJ [20,21,22]. Hence, an overexpression of cytoplasmic X-proteins and a delayed RIANS may cause radiosensitivity because some DSB are not recognized or repaired, and cancer because of DSB non-recognized by NHEJ may be misrepaired [4]. In the RIANS model, the mutated protein responsible for each syndrome associated with delayed RIANS may elicit two impaired functions: one in the nucleus or cytoplasm, as a single mutated protein, and the other, as an overexpressed cytoplasmic ATM substrate. These two impaired functions may explain impaired DSB signaling and repair defects on one hand and lack of cell cycle checkpoint arrest on the other hand [20].

**Figure 2:**
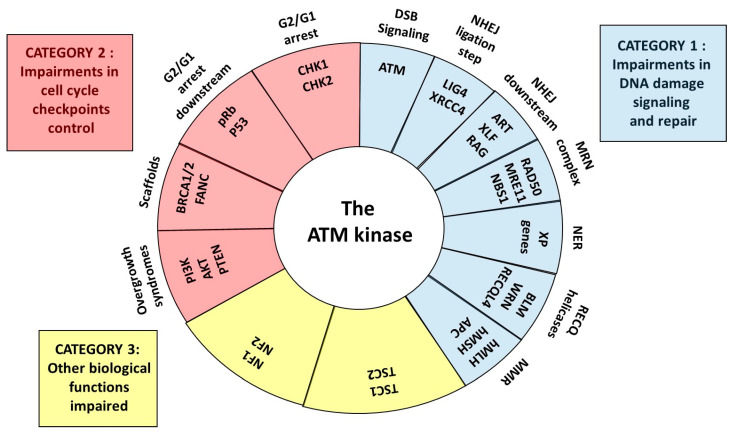
Schematic view of the central role of ATM as a kinase and partner for the proteins whose mutations cause cancer and radiosensitivity syndromes. Three categories can be defined: syndromes due to some impairments in DNA damage signaling and repair, some impairments in cell cycle checkpoint control and to other impaired biological functions.

The RIANS model has been validated in a subset of cancer and radiosensitivity syndromes [8,13,23,24]. This review aims to better identify and understand the molecular and cellular features of the major cancer syndromes associated with radiosensitivity by successively examining the syndromes related to impairments of DNA damage signaling and repair (category 1), impairments of cell cycle checkpoint arrest control (category 2), and the other syndromes for which impairments of both DNA damage signaling and repair and cell cycle checkpoint arrest control are not obvious (category 3) (Figure 2). For each syndrome described, the potential role of the ATM kinase and the RIANS, the presence of SQ/TQ domains potentially phosphorylated by ATM, and the cytoplasmic forms of the mutated proteins will be discussed to provide a novel and unified mechanistic model for both cancer susceptibility and radiosensitivity (Figure 2).

## 2. Diseases of DNA Damage Repair and Signaling

### 2.1. Mutations of the ATM and ATR Kinases

The phosphatidylinositol-3 (PI3) kinases are the components of a large family of enzymes involved in various cellular functions such as cell proliferation, survival and signaling of DNA damage, through their capacity to phosphorylate their substrates [25]. Among them, the ATM and ATR kinases are the two major actors [15,26,27].

The *ATM* mutations play a major role in DNA break recognition, repair and signaling but also in the cell cycle checkpoint control. Homozygous *ATM* mutations cause ataxia telangiectasia (AT), the human syndrome associated with the highest radiosensitivity [1,28,29]. AT was described for the first time by Syllaba and Hennen in 1926 and by Madame Louis Bar in 1941 [30,31]. AT is characterized by cerebellar ataxia with severe prognosis oculocutaneous telangiectasia, a deficient synthesis of immunoglobulins IgA, IgE and IgG2 and a strong predisposition to certain cancers, notably leukemias and lymphomas [32,33,34]. AT is also associated with a spontaneous reorganization of chromosomes (10% of metaphases elicit aberrations on chromosomes 7 and 14) [35]. In the United States and Great Britain, the incidence of AT is estimated to be about 1/100,000 [36]. AT cell lines are systematically characterized by hyper-radiosensitivity [28]. Other radiobiological features of AT cells include numerous chromosomal aberrations, lack of control of the G1 cycle and inhibition of DNA synthesis (called radio-resistant synthesis). No hypersensitivity to UV has been observed in AT patients [32,37,38,39,40]. The *ATM* gene has been cloned and sequenced in 1995 [29,41]. As evoked above, the ATM kinase preferentially phosphorylates the SQ/TQ domains in response to IR [19]. Most of the homozygous *ATM* mutations lead to complete inactivation of the protein [42]. For example, cells from AT patients either do not show γH2AX foci or show a small number of tiny γH2AX foci, suggesting an absent or impaired RIANS [18].

About 15% of AT patients, with so-called variants, suffer from mutations that lead to a less severe pathology, less marked clinical signs, and a life expectancy of 50–60 years (vs. less than 30 years for “classical” AT patients). Such mutations do not concern the kinase domain of ATM [43,44]. Heterozygous carriers (ATH) represent about 1% of the whole population and elicit an increased risk of breast cancer [45], although this epidemiological feature is still debated [46,47,48]. ATH cells may be not more radiosensitive than cells from radioresistant, apparently healthy individuals, although such a hypothesis remains to be confirmed [49,50,51,52]. Altogether, this short review shows that homozygous “classical” and variant *ATM* mutations are associated with both impaired DNA damage signaling and repair and a lack of cell cycle checkpoint control.

The ATR kinase also belongs to the PI3K family and elicits an ATM-like structure with the same affinity for the SQ/TQ domains as ATM. However, ATR is activated by UV, SSB and BD rather than IR and DSB, such as ATM [15,27,53]. ATR may be more essential than ATM for cell viability since only punctual mutations of *ATR* have been observed: such *ATR* mutations cause Seckel syndrome (or microcephalic primordial dwarfism (SCKL)) [54,55,56]. SCKL is associated with severe microcephaly, bird-headed dwarfism, growth and mental retardation. However, literature data suggest that SCKL is not associated with a strong radiosensitivity such as that observed in AT cells [56]. Regarding the susceptibility to cancer, it seems that it is not a major feature of SCKL, although some cases of leukemias have been reported [56]. Lastly, it has been shown that mutations of the pericentrin *PCNT* gene cause a SCKL-like syndrome, suggesting a possible interaction between the PCNT and ATR proteins. *PCNT* mutations have been associated with high cancer risk. However, the level of radiosensitivity potentially associated with *PCNT* mutations needs to be documented [57]. Hence, the literature data suggest that further investigations are needed to better document that SCKL and SCKL-like syndromes can be considered as cancer and radiosensitivity syndromes.

### 2.2. Non-Homologous End-Joining Diseases

#### 2.2.1. Mutations of Ku and DNA-PKcs Genes

As said above, the non-homologous end-joining (NHEJ) pathway is the most predominant DSB repair pathway in G0/G1 mammalian cells [58,59]. Historically, as opposed to homologous recombination (HR), another DSB repair pathway, the term “non-homologous” has been added to “end-joining” to give “non-homologous end-joining” [16]. As explained in another review, this term has produced confusion since the notion of strand homology has no sense in the G0/G1 phase (what would “homologous” end-joining mean?) [4].

The end-joining consists in a ligation of both broken DNA ends. The Ku80 and Ku70 proteins bind to DNA to form the Ku heterodimer. The Ku heterodimer slides on DNA and stops at the broken ends: a third protein, DNA-PKcs, is then recruited and the trimeric complex, named DNA-PK, and acts as a serine-threonine kinase [60]. When activated as kinases (generally after an oxidative stress), ATM and DNA-PK can phosphorylate certain protein substrates such as γH2AX, which triggers the formation of nuclear γH2AX foci at the DSB sites. As evoked above, the formation of nuclear γH2AX foci is considered as the earliest recognition step of DSB managed by NHEJ [61,62]. After this step, ligase IV (LIG4) and XRCC4 proteins are recruited at the DSB sites, and broken DNA ends are joined. This is the ligation step [60]. The biological role of the DNA-PK components (Ku70, Ku80, DNA-PKcs) is so crucial for cell viability that no human syndrome is caused by their mutations. However, in rodents, some Ku and DNA-PKs mutants exist, and they are characterized by a severe defect of DSB repair and hyper-radiosensitivity [63]. In humans, only the glial tumor line MO59J shows a mutation of *DNA-PKs* [64,65], and only one patient has been identified with a *DNA-PK* mutation that does not concern the kinase domain [66,67]. In some cases of lupus erythematosus, an autoimmune disease, the expression of Ku proteins is generally low. However, no systematic link between lupus and radiosensitivity has been established yet, probably because the Ku protein is so abundant that a decreased expression does not significantly affect its role in the response to IR [68]. Hence, considering their importance in cell viability, no cancer and radiosensitivity syndrome associated with mutations of the early actors of NHEJ (namely the components of the DNA-PK complex) have been described yet.

#### 2.2.2. Mutations of the LIG4 and XRCC4 Genes

In 2003, from the case of a patient suffering from a lymphoma who succumbed to its radio-chemotherapeutic treatment [69,70], O’Driscoll et al. have defined a human syndrome associated with *LIG4* mutations, characterized by high radiosensitivity, immunodeficiency, strong pancytopenia, growth retardation and dysmorphic facial features [54]. To date, in addition to this historical case, about forty cases of patients holding *LIG4* mutations have been described [71,72].

As evoked above, the XRCC4 protein forms a complex with LIG4. *XRCC4* mutations may cause microcephalic primordial dwarfism associated with cardiomyopathy but not with immunodeficiency nor predisposition to any malignancy [73,74]. About 15 cases of *XRCC4* mutations have been reported in humans. The first was described in 2014 [75].

In the frame of the RIANS model, can the LIG4 and XRCC4 proteins serve as X-proteins? The LIG4 and XRCC4 proteins hold seven and one SQ/TQ domain, respectively, but no ATM phosphorylated form of these two proteins has been described yet. Some cytoplasmic forms of LIG4 have been reported in response to specific viral infections [76] and the LIG4 protein has been shown to regulate the nuclear localization of XRCC4 [77,78], suggesting that XRCC4 may potentially interact with ATM in the cytoplasm in the case of specific mutations of *LIG4*. However, considering the limited number of LIG4 and XRCC4 patients, further experiments are needed to verify such hypotheses.

#### 2.2.3. Mutations of Art, XLF, 53BP1, RAG1 and RAG2 Genes

The Artemis protein, encoded by the *Art/DCLRE1C* gene, is a DNA exo/endonuclease that acts with DNA-PK to prepare broken DNA ends for ligation [79,80]. Such a step is important, not only for DSB repair via NHEJ but also for the V(D)J recombination process, required for immunoglobulins production. The hypomorphic (homozygous or compound heterozygous) mutations of *Art* cause Artemis syndrome, a human severe combined immunodeficiency associated with a moderate radiosensitivity but a variable predisposition to lymphoma [81,82,83,84,85]. With 10 SQ/TQ domains, the Artemis protein was found to be a major ATM phosphorylation substrate in response to IR that may serve as a regulator of the G2/M cell cycle checkpoint [86]. However, the subcellular localization of the Artemis protein when mutated needs to be documented.

Another important but non-essential NHEJ protein, XLF/Cernunnos, has been identified. Similar to Artemis, XLF/Cernunnos also acts downstream DNA-PK [87,88,89]. Hypomorphic (homozygous or compound heterozygous) mutations of this gene cause Cernunnos syndrome that is associated with mental retardation, microcephaly, strong lymphopenia, and severe immunodeficiency [90]. XLF interacts with XRCC4-LIG4 complex, which stimulates the LIG4 ligase activity by helping to align broken DNA ends. To date, only five Cernunnos syndrome patients have been identified. Similar to Artemis, Cernunnos syndrome is associated with moderate radiosensitivity and a variable predisposition to lymphoma [87,88,89,90]. With four SQ/TQ domains, the XLF/Cernunnos protein was also found to be an ATM phosphorylation substrate in response to IR [91]. The Akt kinase was shown to phosphorylate XLF, which triggers its dissociation from the LIG4-XRCC4 complex and its cytoplasmic relocalization [92]. Further investigations are needed to identify the role of ATM in the cascade of phosphorylation between Akt, XLF, XRCC4 and LIG4.

The 53BP1 protein has been hypothesized to act as a NHEJ protein, although its role is still poorly understood. The 53BP1 protein forms nuclear foci after irradiation, such as many repair proteins cited in this review [93]. The absence of 53BP1 nuclear foci in a patient who showed strong immunodeficiency, dysmorphic aspects, intellectual difficulties and radiosensitivity comparable to Artemis syndrome has been reported. This case was at the origin of the definition of the RIDDLE syndrome (radiosensitivity, immunodeficiency, dysmorphic features and learning difficulties). However, further investigations have documented the molecular features of RIDDLE syndrome that appeared to be caused not by mutations of *53BP1* but by mutations of the ubiquitin-ligase RNF168 [93,94,95]. Hence, while loss of *53BP1* function has been observed in some tumors, no syndrome associated with *53BP1* mutations has been defined yet [93,94,95].

Finally, in this list of syndromes associated with NHEJ impairments, Omenn syndrome (OS) was found to be caused by mutations of the *RAG1* and *RAG2* genes but also by certain mutations of Artemis [96,97]. OS is a severe immunodeficiency syndrome associated with erythroderma, hepatocellular carcinoma, splenomegaly, lymphadenopathy, and some alopecia. OS patients have a low or total absence of B lymphocytes. The few radiobiological studies about OS suggest that the radiosensitivity of OS is similar to that associated with Artemis and Cernunnos syndromes [98,99]. Phosphorylation of RAG1 or RAG2 by ATM has been described [100] but the subcellular localization of the mutated RAG1 and RAG2 proteins needs to be better documented before considering RAG proteins as potential X-proteins.

#### 2.2.4. NHEJ Impairments: Immunodeficiency Rather Than Radiosensitivity?

Unlike with the early actors of the NHEJ pathway, the mutations of the downstream NHEJ actors such as *Art*, *XLF*, *RAG1*, *RAG2* cause a moderate radiosensitivity rather than hyper-radiosensitivity, probably because their functions are redundant during the NHEJ process. When cancer proneness was also reported, all the proteins concerned were found to be phosphorylation substrates of ATM. Since the NHEJ actors are required for a normal V(D)J recombination process, unlike for DSB repair, the major clinical features of the NHEJ impairments described above is severe immunodeficiency rather than severe radiosensitivity. Furthermore, considering the importance of the role of NHEJ actors in lymphocytes, the NHEJ impairments are generally associated with a high risk of leukemia/lymphoma rather than any other cancer type. Most of the mechanistic NHEJ models proposed in the literature do not integrate ATM kinase, while this protein acts upstream the NHEJ actors that serve as phosphorylation substrates, and *ATM* mutations lead to the lack of recognition of a great majority of RI DSB [4,5,16].

## 3. Recombination Repair Diseases

### 3.1. Mutations of the RAD51 and RAD52 Genes

In general, recombination consists in replacing the damaged DNA sequence either by the identical sequence of the homologous chromosome (homologous recombination, HR) [101] or by a sequence taken randomly (illegitimate or non-homologous recombination, NHR), the most frequent ones being the AGCT (*AluI*) sequences [102,103,104]. While a functional HR is required for meiosis and mitosis, and more generally, in proliferating organisms (e.g., bacteria, yeasts), HR is nearly absent from quiescent cells [101]. Although many studies suggest that the RAD51-RAD52 multimeric complex is essential for the recognition of DSB by HR, the subsequent DNA strand exchange process is still poorly understood. Similar to the Ku heterodimer, the RAD52 protein, as a multimeric ring, was hypothesized to slide along the DNA and stop at the DSB sites. At the DSB sites, RAD51 may associate with RAD52, and its phosphorylation by tyrosine kinases may activate both its nuclease function and its change of shape as a filament [101,102,103,104]. While the RecA protein is considered as the human RAD51 homolog and is essential for the exchange of DNA strands in bacteria, the *RecA* sequence is however much smaller than that of *RAD51* and does not hold any endonuclease site such as RAD51 [105]. Furthermore, resolvases and the complex process of DNA strand exchange (Holliday junctions) remain misunderstood in humans [106]. As evoked for the upstream NHEJ proteins, the mutations of the upstream HR actors lead to the absence of viability, and no corresponding human syndrome exists. Lastly, yeast strains carrying *Rad52* mutations are among the most hyper-radiosensitive ones, confirming the importance of HR for proliferating organisms and the differences existing between micro-organisms and mammalians [107].

#### 3.1.1. The Hyper-Recombination Process—At the Origin of Carcinogenesis?

Many proteins other than RAD51 and RAD52 are involved in the recombination process and ensure the stability of the genome via multiprotein complexes. This is particularly the case of scaffold proteins such as BRCA1, BRCA2, and FANC, whose mutations combine cancer predisposition and lack of control of the cell cycle checkpoints (the consequences of their mutations will be discussed in the next chapter). In addition, the mutations of these proteins cause a lack of control of recombination (also called hyper-recombination). The hyper-recombination process is responsible for the production of additional and misrepaired DNA breaks [108,109,110]. In other terms, hyper-recombination results in an accumulation of errors and numerous spontaneous breaks, notably through the impairment of nucleases function [108,109,110]. Hyper-recombination is a common feature of all the syndromes associated with a high risk of cancer. In the frame of the model of carcinogenesis proposed by Weinberg, hyper-recombination and the resulting gene mutations may serve as the endogenous initiation step required for cell transformation and tumorigenesis [111].

#### 3.1.2. Mutations of the RAD50-MRE11-NBS1 Complex

The RAD50, MRE11 and NBS1 proteins form a complex involved during the several steps of DNA damage response from DNA damage recognition to assembly repair complexes, and mutations of proteins constituting this complex are associated with neurological syndromes with tumorigenic potential. Furthermore, the MRE11 endonuclease activity was shown to strongly depend on the integrity of the RAD50-MRE11-NBS1 complex [112,113,114].

The mutations of *NBS1* cause Nijmegen’s syndrome (NBS), first described in the 1980s [115]. NBS was long considered as a variant form of the AT syndrome, but with a lower radiosensitivity [116,117]. Microcephaly, small stature, mental retardation, high lymphoma susceptibility and immunodeficiency are the main clinical manifestations of NBS [115]. NBS patients elicit neither ataxia nor telangiectasia [116]. *NBS1*-mutated cells are characterized by chromosomal instability, and their radiosensitivity may be considered as hyper-radiosensitivity even if it is systematically lower than that observed in AT cells [4,116,117,118]. *NBS1*-mutated cells show a lack of cycle arrest in G1 [116]. Two groups of complementation V1 (Berlin syndrome) and V2 (Nijmegen’s syndrome) were described with the same absence of cycle arrest in G1 [119,120]. However, some studies have reported that there is only one *NBS1* gene and that it is located on chromosome 8 [121,122,123]. The NBS1 protein (also called nibrin) may serve as a phosphorylation substrate of ATM with seven SQ/TQ domains [124]. As a scaffold for the RAD50-MRE11-NBS1 complex, in NBS cells, the NBS1 protein is absent in NBS cells, and MRE11 and RAD50 are cytoplasmic, suggesting that these two proteins may serve as X-proteins in the case of mutations of the *NBS1* gene [125] (see also below).

Mutations of *MRE11* have been initially identified in three patients whose fibroblasts have been found radiosensitive and deficient in DNA damage repair. The radiosensitivity associated with mutations of *MRE11* is lower than with *NBS1* mutations [126]. Historically, the first identified family with homozygous mutations of *MRE11* showed similar clinical features to AT but with less pronounced intensity. The associated syndrome has been therefore called Ataxia–Telangiectasia-Like Disorder (ATLD) [126]. After identifying other families, ATLD is now considered as a neurological syndrome with radiosensitivity comparable to NBS but associated neither with immunodeficiency nor with high susceptibility to cancer [127,128,129]. With seven SQ/TQ domains, MRE11 is a phosphorylated substrate of ATM that is localized in both the cytoplasm and nucleus and provides nuclear foci after exposure to IR in an ATM-dependent manner. We have shown that cancer syndromes are generally associated with the formation of MRE11 foci early (some minutes) after irradiation while aging syndromes are generally associated with the formation of late foci (24 h) after irradiation [5].

Finally, the case of a patient showing a mutation of *RAD50* has been described in 2009 with microcephaly, mental retardation, a bird face, and a small stature [130]. This *RAD50*-mutated patient developed a malignant lymphoma at the age of 23 without severe immunodeficiency. The cells from this patient elicited radiosensitivity similar to those observed with NBS. Such a syndrome has been called Nijmegen Breakage Syndrome Like-Disorder (NBSLD) [130]. With seven SQ/TQ domains, the RAD50 protein is phosphorylated by ATM at Ser-635 that plays an important adaptor role in signaling for cell cycle control and DNA repair [131,132]. However, in the cells from the single NBSLD case, the MRE11 protein predominantly appeared cytoplasmic, and the RAD50 protein was absent [130].

Altogether, this brief review about the mutations of the RAD50-MRE11-NBS1 complex reveals that all the components of the complex are some ATM phosphorylation substrates, and the mutation of one component may lead to the cytoplasmic localization of at least one of the other components of the RAD50-MRE11-NBS1 and to at least the possibility of sequestrating ATM and preventing rapid RIANS.

#### 3.1.3. Mutations of the Nucleotide and Base Excision Repair Genes

The single-strand annealing (SSA) pathway has been defined only in vitro with very specific sequences (short DNA sequences in which some reporter genes have been placed very close (few hundred bases) to each other) [133,134]: in such a limited and specific DNA sequence system, even a random phenomenon can lead to a faithful repair. Hence, caution must be taken about any in vivo extrapolation of the SSA phenomenon since coding sequences are generally much more spaced and the genome is much longer than the sequences used for the investigations about SSA. Lastly, few proteins have been considered specific to SSA, which relativizes again the existence of such a repair pathway in vivo, especially in response to IR [4].

It has been shown that ligase III, PARP, XRCC1 are the major proteins involved in the base excision resynthesis (BER) [135,136]. However, no genetic syndrome has been associated with mutations of the BER proteins in humans. Such a situation is similar to that discussed above with the Ku heterodimer and with the RAD51 and RAD52 proteins: XRCC1, PARP, ligase III, and DNA polymerase β are proteins required for DNA damage recognition (here BD recognition). They may be so abundant and essential for survival that their mutations systematically lead to a loss of viability: the homozygous mutations of these three genes are lethal at the embryonic state. However, the literature regularly reports diseases associated with BER defects or impairments, but these are generally either the syndromes already mentioned whose mutated genes are not directly involved in BER, or else polymorphisms [136]. Lastly, PARP inhibitors are currently used in the treatments of *BRCA1/2*-mutated tumors in which DNA breaks accumulate until cell killing [137].

Unlike SSA and BER, the nucleotide excision resynthesis (NER) pathway involves many proteins acting from BD recognition to final DNA sequences polymerization, whose mutations may cause human syndromes associated with cancer, radiosensitivity or even photosensitivity. Particularly, the mutations of some *XP* genes involved in the transcription factor II H (TFIIH) complex, essential for a functional NER, can cause xeroderma pigmentosum syndrome (XP). XP is dispatched in several groups of complementation [138,139]. Some XP proteins are involved in the NER as endonucleases, helicases, or oriented polymerases. This is notably the case of *XP-A* to *XP-G XP-G*, whose mutations are linked to photosensitivity, neurodegeneration and/or brain or skin cancer [139,140,141]. In addition, some complementation groups such as XPD may be also associated with moderate radiosensitivity, suggesting some role in DSB repair and signaling [23,142]. All the mutations of *XP-A* to *XP-G* genes may cause misrepaired BD, SSB and/or DSB [4]. All the *XP-A* to *XP-G* genes hold SQ/TQ domains and show cytoplasmic forms when mutated, suggesting that ATM may phosphorylate and interact with them in the cytoplasm. For example, some specific mutations of XPD were shown to sequestrate ATM in the cytoplasm after exposure to IR and lead to cancer proneness and radiosensitivity [23].

Often necessary during or after the DNA strands exchange process, the RECQ helicases are inextricably linked to the maintenance of genome integrity. The RECQ family contains three proteins identified in humans, including WRN, BLM, RECQL4 [143,144]. Mutations of *BLM*, *WRN* and *RECQL4* cause Bloom (*BLM*), Werner (*WRN*) and Rothmund–Thompson (*RTS*) syndromes, respectively. The BLM, WRN and RECQL4 proteins show nuclease domains, which support their involvement in hyper-recombination processes [145,146,147]. In addition to growth disorders and accelerated aging, these three syndromes have in common a strong predisposition to sarcoma. With regard to aging and predisposition to early senescence, it can be hypothesized that an instable helicase–endonuclease complex would be responsible of the generation of spontaneous breaks that may promote a senescence phenomenon [145,146,147]. All these syndromes are associated with significant but moderate radiosensitivity [1].

The WRN and BLM proteins hold SQ/TQ domains and have been shown to be phosphorylated by ATM [148,149]. The WRN and BLM cells show cytoplasmic forms [150,151]. With regard to RECQL4 cells, we recently pointed out the existence of the SQ/TQ domain, the cytoplasmic forms of some mutated RECQL4 proteins, and the delay of RIANS.

## 4. Mutations of Mismatch Repair Genes

Human non-polyposis hereditary colon cancers (HNPCC) syndromes are often grouped under the name of Lynch syndrome (LS), even though some physicians distinguish both [152,153,154]. HNPCC syndromes are caused by mutations of DNA mismatch repair genes (MMR) [155]. MMR is a DNA damage repair and signaling pathway that manages erroneous insertions or deletions of bases during DNA replication (S phase) and recombination (mitosis) [156]. Such a pathway is therefore particularly active in proliferating cells and tissues. By highlighting the biases raised by the extrapolation from micro-organisms to mammalians evoked above, it is noteworthy that many MMR genes found in yeasts do not exist in humans. Mutations of the MMR *hMLH1*, *hMSH2*, *hMSH6* and *hPMS2* genes are responsible for many forms of HNPCC [152,153,154,155]. HNPCC show a high susceptibility to colon cancer but also to endometrial, ovarian, stomach, small intestine, liver, upper urinary tract, brain, and skin cancers [153,157]. HNPCC are generally characterized by chemosensitivity and moderate radiosensitivity. However, some cases of severe radiosensitivity have been reported but always in the context of Turcot syndrome (TS), a pathology often associated with the mutation of *hMSH2* [158]. Intestinal pathologies generally associated with mutations of the *APC* gene may also be associated with *MMR gene* mutations: familial recto-colic polyposis and attenuated forms of familial recto-colic polyposis, Gardner syndrome (GS) and Turcot syndrome [154,158]. The last two syndromes are associated with other tumors such as osteomas, fibroids, lipomas and thyroid or adrenal tumors for GS and central nervous system tumors for TS [154,158]. Although polyposis is generally associated with mutations of the *APC* gene, mutations of *hMSH2* can also cause TS as mentioned above. Both GS and TS are associated with significant but moderate radiosensitivity. However, the biological functions of the *APC* gene are still unknown, but some studies have reported a role of *APC* in the regulation of mitosis microtubules, which cannot explain the radiosensitivity observed in quiescent cells derived from these syndromes [1,158].

The hMLH1, hMSH2, hMSH6, hPMS2 and APC proteins hold SQ/TQ domains, show cytoplasmic localization when mutated and can interact with ATM [159,160].

## 5. Diseases of Cell Cycle Checkpoint Control

### 5.1. A Lack of Control of the Cell Cycle Checkpoint, Another Requirement for Carcinogenesis?

In the frame of the most current models of carcinogenesis, including the initiation/promotion/progression model, one mutation in one cell or even one mutation in several cells does not necessarily lead to cell transformation and cancer [111]. An amplification of the number of cells holding mutations is therefore required to ensure the formation of a pre-tumor. Such amplification may occur when the cell cycle checkpoints are impaired. Let us review the major diseases of the cell cycle checkpoint control.

### 5.2. Overgrowth Syndromes

The PI3K kinase is composed of an 85 kDa regulatory subunit and a 110 kDa catalytic subunit. Among the numerous variants of these subunits, the p110α one, called PI3KCA, has been shown to be involved in the control of cellular proliferation. Some somatic mosaic mutations of the *PI3KCA* gene are associated with overgrowth syndromes called PI3KCA-related overgrowth spectrum (PROS) syndromes [161,162,163,164,165]. Some cases of cancers have been reported in the PROS patients [164,165]. All these syndromes are associated with overgrowth malformations in skin, vasculature, bones or brain tissues due to somatic mosaic heterozygous mutations leading to overactivity of the PI3K kinase [161,162,163,164,165]. ATM and PI3K kinases have been shown to interact and activate in response to genotoxic stress [161,166,167]. Recently, we have shown that PROS syndromes are associated with radiosensitivity and radiosusceptibility: some mutations of the *PI3KCA* gene lead to the over-expression of cytoplasmic forms that, as substrates of ATM, result in a delayed RIANS [161].

Proteus syndrome, which does not belong to the PROS syndromes family, is characterized by tissue overgrowth, and hyperplasia of multiple tissues may also be associated with high susceptibility to the development of tumors. Proteus syndrome is caused by somatic activating mutations of the *AKT1* gene, which triggers activation of the PI3K-AKT pathway [168]. The pleckstrin homology domain of AKT binds to the cellular membrane via its affinity for PI molecules phosphorylated by PI3K, which stimulates cell proliferation and invasiveness. Cytoplasmic ATM was found to be an upstream activator of AKT1, and both proteins are involved in a common pathway with PI3K that promotes cell proliferation when altered or hyperactivated [169,170].

The PTEN protein is a phosphatase responsible of the dephosphorylation of PI molecules, which inhibits the PI3K-AKT signaling pathway described above [171,172]. In the case of *PTEN* mutations, the PI3K-AKT signaling pathway is activated, which stimulates cell proliferation and invasiveness [171,172,173,174,175]. Inherited mutations of the *PTEN* gene notably cause Cowden disease associated with a high risk of developing breast cancer [176]. The *PTEN*-mutated cells were found to be sensitive to radiation [177]. ATM is known to phosphorylate PTEN in the cytoplasm, which triggers its translocation from the cytoplasm to the nucleus in response to oxidative stress [178].

### 5.3. Mutations of the CHK1 and CHK2 Genes

Checkpoint kinases 1 and 2, namely CHK1 and CHK2, are serine/threonine kinases that coordinate cell cycle response to genotoxic stress and are generally activated by the phosphorylation of both ATM and ATR kinases [15,179,180]

*CHK1* is particularly required for G2/M arrest in response to IR [15,180]. However, while *CHK1* gene overexpression (as its non-phosphorylated form) has been reported in several tumor models, no germline *CHK1* mutation has been detected in any cancer syndrome. Conversely, heritable mutations within the *CHK1* C-terminal regulatory domain have been recently shown to cause female infertility in humans through the blockage of oocytes in their first mitosis [180].

Unlike CHK1, CHK2 phosphorylated by ATM is required for ensuring G1 arrest in response to IR [15,179]. Again, unlike *CHK1*, several *CHK2* mutations have been observed in different types of cancers, including prostate, colon, lung, thyroid and mainly breast cancers. Hence, to date, *CHK2* is considered as a tumor suppressor gene, a phosphorylation substrate of ATM [181]. Overexpression and cytoplasmic localization of CHK2 was observed in a large subset of tumors and are associated with genomic instability and high levels of DNA damage [182,183].

### 5.4. Mutations of the BRCA1, BRCA2, FANC Genes

While the BRCA1 and BRCA2 proteins have almost the same acronyms, they do not share identical sequences or domains [184]. The BRCA1 protein is a large protein (220 kDa), and two major structural domains have been identified: a RING domain in its N-terminal region, and a tandem of two BRCT (BRCA1 C-terminal) domains [185,186]. This last domain is found in many proteins involved in DNA damage repair (e.g., LIG4) and cell cycle checkpoint control (e.g., p53) [187]. Furthermore, BRCA1 holds 13 SQ and 3 TQ domains that are specifically phosphorylated by ATM and ATR kinases [19]. The phosphorylation of BRCA1 by ATM in response to IR leads to the formation of nuclear foci [188,189]. Except for the three identified domains, RING, BRCT and the SQ/TQ cluster, no other functional sites such as kinase, ligase, nuclease, etc., have been identified in the *BRCA1* sequence [190]. The BRCA1 protein is localized both in the nucleus and cytoplasm and acts as a scaffold protein for a multitude of ATM phosphorylation substrates [188,189]. Homozygous mutations of *BRCA1* are not viable in humans. Heterozygous mutations of *BRCA1* lead to inherited breast cancer syndromes but are also seen in about 18% of ovarian cancers and represent a significant factor of risk for other cancers such as prostate cancers [191,192,193,194]. Mutations in the C-terminal BRCT domains of the BRCA1 protein result in cytoplasmic mislocalization [195,196,197].

BRCA2 has a molecular weight of 384 kDa and is a protein larger than BRCA1. BRCA2 contains BRC domains different from the BRCT domains observed in the *BRCA1* sequence. Similar to BRCA1, the BRCA2 protein serves as a scaffold and has many protein partners, including the ATM kinase [191]. Similar to *BRCA1*, homozygous mutations of *BRCA2* do not exist in humans since they cause lethality at the embryonic state. Heterozygous mutations of *BRCA2* are the cause of inherited ovarian cancers and male breast cancers [110,190,198].

The BRCA1 and BRCA2 proteins are essential for the action of RAD51 and RAD52 in active HR in the G2/M phase [189]. Interactions between BRCA1 and MRE11 have also been described, reinforcing the hypothesis that BRCA1 may participate in both HR and NHR processes [199,200]. Most *BRCA1* and *BRCA2* mutations confer a moderate radiosensitivity in G1 comparable to that observed in the case of *MRE11* mutations [201]. Some mutations of *BRCA1* and *BRCA2* are also associated with high chemosensitivities, particularly to alkylating agents such as cis-platinum [201,202]. However, some studies about the RI cascade of phosphorylations of ATM substrates show that BRCA1 and BRCA2 should be considered more as cell cycle checkpoint proteins than as DNA repair proteins since the kinetics of RI phosphorylation of BRCA1/2 proteins are slower than the molecular events involved in DNA damage recognition and repair [15].

The Fanconi anemia (FA) complementation group (FANC) gathers 14 FANC proteins that are involved in post-replication repair and in cell cycle checkpoint control [203,204]. Most of them interact with BRCA1/2 proteins and ATM (FANCD2 and BRCA2 are the same protein) [110,205]. Furthermore, such as the BRCA1/2 proteins, the FANC proteins have no active domain for ensuring a specific enzymatic function. Mutations of the FANC proteins cause Fanconi anemia (FA) syndrome that was first described by the Swiss pediatrician Guido Fanconi in 1927 [206,207,208]. FA is one of the major hereditary syndromes of spinal cord failure. It is often associated with congenital malformations (including microcephaly), growth defects (small size), skin disorders (café-au-lait spots) and generally progresses to aplasia or leukemia. The predisposition to FA-related cancer is not limited to lymphocytes, but also extends to breast cancer. Rather characterized by their chemosensitivity, cells from FA patients show low but significant radiosensitivity [206,209,210,211]. Although the FANC proteins hold numerous SQ/TQ domains, the phosphorylation of FANC by ATM has been described only for FANCD2/BRCA2 [191,209]. In addition, since nearly all the FANC proteins show cytoplasmic forms, whether mutated or not, mutations of the *FANC* gene may lead to a sequestration of ATM by FANC proteins. However, further experiments are needed to confirm this hypothesis. 

### 5.5. Mutations of the RB1 and P53 Genes

The pRB protein, a product of the *RB1* gene, acts as a negative regulator of the cell cycle, notably by blocking cells in G1 phase through its non-phosphorylated form. In proliferating cells, the cyclin-dependent kinase complexes phosphorylate pRB, which liberates the E2F transcription factor and favors the transition to S phase [212,213]. Heterozygous germline *RB1* mutations cause retinoblastoma syndrome (RB), a rare pediatric disease associated with tumors in retina cells [214,215,216,217]. The pRB protein is also a phosphorylation substrate of the ATM kinase, and we have shown that skin fibroblasts from RB patients elicit moderate but significant radiosensitivity associated with delayed RIANS caused by the cytoplasmic overexpression of some mutated pRB proteins [24].

The p53 protein is the most documented of the human transcription actors and is involved in the cell cycle checkpoint and in some specific cellular death pathways [218,219,220]. Again, from studies about the RI cascade of phosphorylations of ATM substrates, p53 cannot be considered as a DNA damage repair protein since its activation appears later than the DNA damage recognition and repair step [15]. Similar to the pRB and BRCA1/2 proteins, p53 is phosphorylated by the ATM kinase in response to IR [15]. While homozygous mutations of the *p53* gene are lethal in the embryonic state, heterozygous mutations of *p53* cause Li-Fraumeni syndrome (LFS), associated with predisposition to rhabdomyosarcoma, but also to multiple cancers such as in muscles, breast, bones, and blood cancers [221,222]. Similarly to pRB, we have shown that skin fibroblasts from LFS patients elicit moderate but significant radiosensitivity associated with delayed RIANS caused by the cytoplasmic overexpression of some mutated p53 proteins [222].

## 6. Cancer Syndromes and the RI ATM Nucleoshuttling Model

Some cancer syndromes may be caused by mutations of genes whose protein products are directly involved either in DNA repair and signaling or in cell cycle checkpoint control. Such diseases therefore raise the question of the molecular mechanisms of carcinogenesis. Let us review the molecular and clinical features of the major ones.

### 6.1. Mutations of the NF1, NF2, TSC1 and TSC2 Genes

The *NF1* gene encodes for the neurofibromin 1 protein, a GTPase-activating protein involved in neural development. Furthermore, neurofibromin was reported to modulate the *Ras*-dependent oncogenic pathways, which may favor abnormal proliferation [223]. However, considering individual specificities, the *NF1* gene cannot be considered as directly involved in cell cycle checkpoint control such as *CHK1* or *CHK2*. The heterozygous mutations of neurofibromin cause neurofibromatosis type 1 (NF1) syndrome associated with *benign* tumors along the peripheral and optic nerves and malignant tumors such as neurofibrosarcomas, astrocytomas, and rhabdomyosarcomas [224,225,226,227,228]. A study has shown that the neurofibromin 1 protein was a substrate of ATM kinase and that cells from NF1 patients elicit a moderate but significant radiosensitivity associated with delayed RIANS caused by the cytoplasmic overexpression of the mutated neurofibromin [13].

The *NF2* gene encodes for the neurofibromin 2 protein (also called schwannomin or moesin–ezrin–radixin (merlin) protein), which is a cytoskeletal protein. Neurofibromin 2 is considered as a scaffold protein linking transmembrane receptors, actors of cell adhesion, small GTPases, mTOR- and PI3K/AKT-dependent pathways proteins [223,229]. Loss of function mutations or deletions in *NF2* genes cause neurofibromatosis type 2 (NF2) syndrome associated with a multiple-tumor-forming disease of the nervous system and notably schwannomas, meningiomas and ependymomas [229,230]. Even if the clinical consequences of the mutations of *NF2* gene strongly suggest that it is a tumor suppressor gene, the biological role of the merlin is not documented enough to consider it as directly involved in DNA damage repair and signaling and/or in the cell cycle checkpoint control. Preliminary experiments in our lab reveal that fibroblasts from NF2 patients show a moderate but significant radiosensitivity (N.F., personal communication). Although the merlin protein holds one TQ domain, no ATM phosphorylation of the merlin protein has been described yet. Conversely, the merlin protein was shown to be cytoplasmic in G0/G1 and mitosis but nuclear in S phase, through the mediation of its phosphorylation by AKT1 in serine 518, hence describing a cell-cycle-dependent nucleoshuttling [231]. Further experiments are needed to examine whether ATM and merlin proteins interact in cytoplasm in cells from NF2 patients.

The *TSC1* and *TSC2* genes encode for the hamartin [232,233] and the tuberin [232,234] proteins, respectively, that interact with a very large multiprotein complex called the tuberous sclerosis complex (TSC). Both proteins are involved in the regulation of cell growth control and the activity of the target of rapamycin (TOR) complex 1 (TORC1). However, their direct role in cell proliferation remains to be more documented [235,236,237,238,239]. Heterozygous mutations of either of the two *TSC1* and *TSC2* genes cause the TSC syndrome [240,241]. TSC is associated with high incidence of angiofibroma, astrocytoma, renal angiomyolipoma and lymphangioleiomyomatosis [232,242]. Recently, it was reported that fibroblasts from TSC patients show a moderate but significant radiosensitivity and that TSC and ATM dynamically interact in response to IR. In cells from TSC patients, hamartin was found overexpressed in cytoplasm and complexed with ATM, therefore causing a delayed RIANS [12].

### 6.2. Requirement of Both Impaired DNA Damage and Cell Cycle Checkpoints

Literature data and this review suggest that carcinogenesis and cell transformation require both misrepaired DNA damage that generates DNA sequence errors and impaired cell cycle checkpoint control that facilitates cell proliferation and therefore errors propagation. These two steps are consistent with the hypothesis of initiation and promotion steps proposed by several oncologists [111]. As evoked above, the cancer and radiosensitivity syndromes described in this review may be therefore classified into three categories (Figure 2):

Category 1: the cancer syndromes caused by mutations of genes directly involved in DNA damage recognition, repair and signaling pathway. Considering the importance of DNA damage recognition and repair in cell viability, these syndromes are generally very rare (prevalence of about 1/100,000), recessive and caused by hypomorphic mutations leading to a partial loss of the function of the gene. However, two subcategories can be considered according to the importance of the mutated gene and the associated radiosensitivity quantified by the cell survival at 2 Gy (SF2). Radiosensitivity can be either extreme (10% < SF2 < 30%) such as *LIG4* syndrome or moderate (30% < SF2 < 60%) such as Artemis syndrome [4,5]. All these gene products are substrates of ATM and may localize in cytoplasm when mutated. The gene mutations may explain misrepaired DNA damage. However, how can the resulting sequence errors be propagated through impaired cell cycle checkpoints?

Category 2: the cancer syndromes caused by mutations of the genes directly involved in the cell cycle checkpoint control. These syndromes are more frequent than the first category (prevalence is higher than 1/40,000), are generally dominant and are caused by heterozygous mutations. One of the most representative examples of such syndromes is Li-Fraumeni syndrome (heterozygous *p53* mutations). Their associated radiosensitivity is moderate but significant (30% < SF2 < 60%) [4,5]. Again, all these gene products are substrates of ATM and may localize in the cytoplasm when mutated. The gene mutations may explain the impairment of the cell cycle checkpoints that lead to the propagation of errors. However, how can the misrepaired DNA damage be generated with such gene mutations? 

Category 3: the cancer syndromes caused by mutations of genes that are directly involved either in DNA damage recognition, repair and signaling pathway or in the cell cycle checkpoint control. The gene mutations that cause these syndromes may be homozygous, heterozygous or de novo, which explains why their prevalence can be lower than the syndromes from Category 2. One of the most representative examples of such syndromes are the PROS syndromes. Their associated radiosensitivity is moderate but significant (30% < SF2 < 60%) [4,5]. Again, all these gene products are substrates of ATM and may localize in the cytoplasm when mutated [12,13]. How can the misrepaired DNA damage be generated and propagated with such gene mutations?

Altogether, these categories raise the question of gene mutations that would lead to two concomitant but distinct molecular and cellular consequences, one in DNA damage recognition and repair and the other in cell cycle checkpoint control. Throughout the RI ATM nucleoshuttling (RIANS) model that is now very documented [20], let us propose a mechanistic model to integrate these three categories of syndromes in a unique explanation.

### 6.3. The ATM Nucleoshuttling Model: A Possible Explanation for Carcinogenesis?

**Figure 3:**
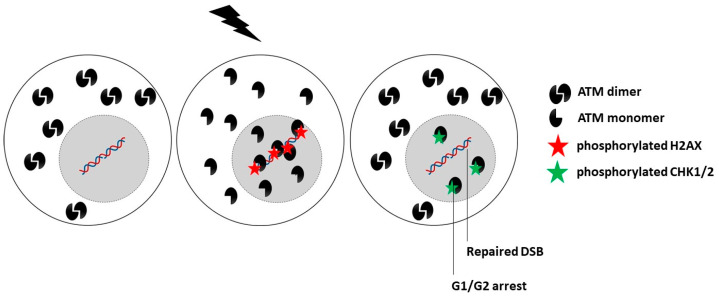
Schematic representation of the RIANS model in radioresistant cells. A rapid RIANS leads to the ATM-dependent recognition of all the DSB induced by IR via the NHEJ pathway, their complete repair, and phosphorylation of the CHK proteins that induce G1/G2 arrest.

While the upstream action of the ATM protein in the predominant DNA damage repair and signaling pathways, notably in NHEJ, suggests a nuclear sublocalization, some authors have reported that ATM is also a cytoplasmic protein [22,243,244,245]. By analyzing hundreds of fibroblast cell lines derived from cancer patients eliciting a large spectrum of post-radiotherapy radiosensitivity, it appears that IR triggers a drastic change in the subcellular localization of ATM. As evoked above, in quiescent cells, after exposure to IR, the cytoplasmic ATM dimers dissociate as ATM monomers and diffuse in the nucleus. Once in the nucleus, ATM monomers phosphorylate H2AX histone protein (γH2AX), which triggers the activation of NHEJ [20,21,22]. However, in the nucleus, ATM monomers also phosphorylate some other ATM substrates such as MRE11 (which limits the nuclease activity of the RAD50–MRE11–NBS1 complex evoked above) and CHK1 and CHK2 proteins (which trigger cell cycle arrest in G2/M and G1, respectively) [15,20]. Nevertheless, in cells that show moderate but significant radiosensitivity, RIANS is delayed by the overexpression of cytoplasmic substrates of ATM, which sequestrate the RI ATM monomers in the cytoplasm. Such a model was confirmed by both immunoblots and proximity ligation assays [20,21,246]. These ATM substrates, called X-proteins, are generally the proteins mutated specifically in the syndrome considered [5,8,18,20,21] (Figure 3). Hence, the RIANS model integrates the hypothesis that two distinct biological functions may be impaired as discussed in Section 1.3. A mutation of an X-protein may lead to: (1) the impairment of its intrinsic biological function as a single protein, and (2) the impairment of some biological functions caused by the X-protein as a cytoplasmic ATM substrate.

Hence, the RIANS model permits to revisit the three categories of cancer syndromes defined above by providing a relevant unified model.

**Figure 4:**
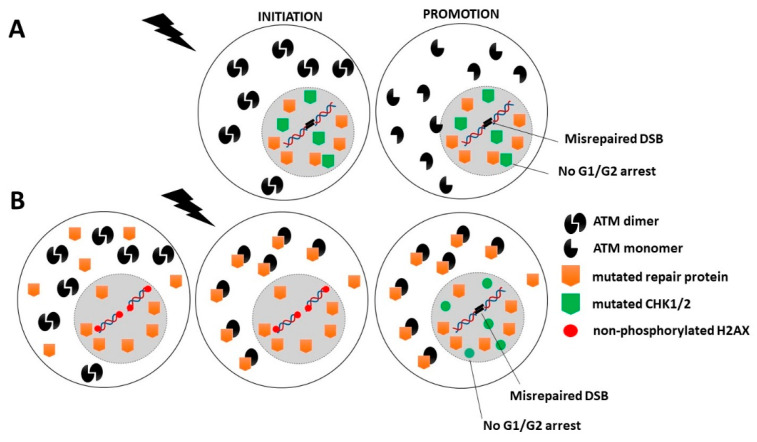
Schematic representation of the RIANS model in cells from Category 1 cancer syndromes. (**A**) First subcategory: Mutated genes are crucial for DNA damage recognition and repair. In this case, the genomic instability is so high that some spontaneous mutations may also affect cell cycle checkpoint control. (**B**) Second subcategory: Mutated genes are involved in DNA damage recognition and repair but are not crucial. The encoded proteins are X-proteins. The RIANS is delayed by the cytoplasmic overexpression of X-proteins, and few ATM monomers enter in the nucleus. Consequently, CHK proteins are not phosphorylated, and therefore G1/G2 arrests are impaired.

Category 1: Two subcategories can be considered. In the first one, mutations of crucial genes directly involved in the DNA damage recognition, repair and signaling that do not result in an embryonic state (such as *ATM* and *LIG4* mutations) produce a level of misrepaired DNA breaks and genomic instability that is so high that it also produces mutations in some other genes involved in the cell cycle checkpoint control. The spontaneous (p14; q11) chromosome exchanges currently observed in *ATM*-mutated cells are a representative example of cytogenetic events that may concern more than one gene [32,33,247]. In other terms, in this category of cancer syndromes, the initiation and promotion steps may occur concomitantly [4,18]. This is notably the case of *ATM-* and *LIG4*-mutations (Figure 4A and Table 1). In the second subcategory, the mutations of less crucial DNA damage recognition and repair genes may result in high cytoplasmic expression of the gene products (as X-proteins). Such expression of X-proteins may lead to delayed RIANS, which limits DSB recognition through incomplete γH2AX phosphorylation and G2/M and G1 arrests through incomplete CHK1 and CHK2 phosphorylation. Hence, in this subcategory, the mutated protein, as an impaired contributor of DNA damage recognition and repair, favors errors formation, while the mutated protein, as an X-protein, delays the RIANS, which favors their propagation via impaired phosphorylation of cell cycle checkpoint control proteins. This is notably the case of some *XPD* mutations (Figure 4B, Table 1).

**Figure 5:**
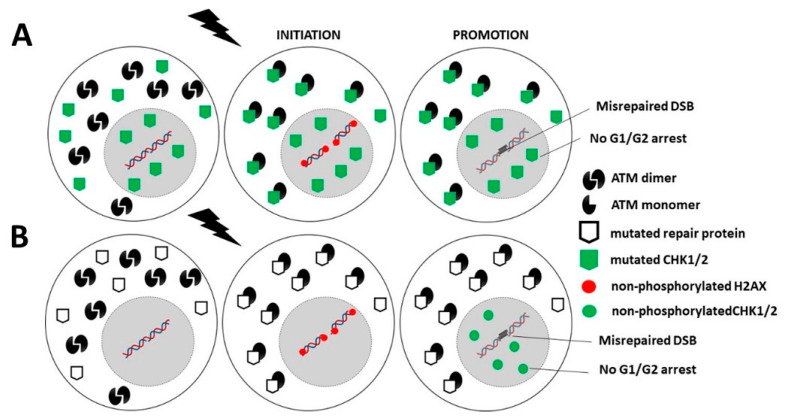
Schematic representation of the RIANS model in cells from Category 2 and 3 cancer syndromes. (**A**). Category 2: Mutated genes are involved in cell cycle checkpoint control. The encoded proteins are X-proteins. The RIANS is delayed by the high cytoplasmic expression of X-proteins, and few ATM monomers enter into the nucleus. Consequently, few DSB are recognized by NHEJ and therefore may be misrepaired. (**B**). Category 3: Mutated genes are directly involved either in DNA damage recognition and repair or in the cell cycle checkpoint control. The encoded proteins are X-proteins. The RIANS is therefore delayed by the cytoplasmic overexpression of X-proteins, and few ATM monomers may enter into the nucleus. Consequently, few DSB are recognized by NHEJ and therefore may be misrepaired. Furthermore, CHK proteins are not phosphorylated, and therefore, G1/G2 arrests are impaired.

Category 2: Mutations of genes directly involved in the cell cycle checkpoint control are generally heterozygous mutations and are associated with a high expression of cytoplasmic forms of the protein. This is notably the case of PI3KCa [161], p53 [201,222], or pRB proteins [24]. These abundant cytoplasmic X-proteins contribute to sequestrate the ATM monomers and therefore lead to a decrease in the flux of ATM monomers entering in the nucleus. Consequently, less DSB are recognized by NHEJ, and the number of misrepaired DSB increases. Hence, in this category, the mutated protein, as an X-protein, delays the RIANS, which favors error formation, while the mutated protein, as an impaired contributor to cell cycle checkpoint control, favors their propagation (Figure 5A and Table 1).

Category 3: Mutated genes that are directly involved either in DNA damage recognition and repair or in the cell cycle checkpoint control may also be associated with high cancer proneness if they are X-proteins. Their heterozygous mutations are responsible for their high expression in the cytoplasm. Thus, as described above, the resulting sequestration of RI ATM monomers contributes to reducing the flux of ATM monomers in the nucleus, to decreasing the DSB recognition by NHEJ and to increasing the number of misrepaired DSB. In addition, a delayed RIANS may also decrease the phosphorylation of CHK1 and CHK2 by ATM in the nucleus, which may impair the G2 and G1 arrests, respectively (Figure 5B, Table 1).

### 6.4. What Are the Limits of the Validity of the RIANS Model?

All the genes whose mutations that cause cancer and radiosensitivity syndromes show SQ/TQ domains: they have been identified or considered as phosphorylation ATM substrates and may present cytoplasmic forms, at least, when mutated, suggesting that the RIANS model may unify the three categories of syndromes defined above. However, the validity of our model should be discussed in three specific conditions of irradiation, at least: the influence of cell cycle, the effect of linear energy transfer (LET) and the influence of low dose, which all may act in cancer proneness and radiosensitivity.

In the frame of the RIANS model, all the DNA damage is supposed to be induced in the G0/G1 phase. In this specific cell cycle phase, the NHEJ repair pathway is predominant. In these conditions, the propagation of errors is mostly conditioned to the impairment of G1/S arrest to reach the G2/M phase. By contrast, the RIANS model is not relevant for the G2/M cells since ATM nucleoshuttling does not exist in this phase. Hence, the model proposed here may be completed with mechanisms describing the fate of the DNA damage when induced during the G2/M phase. In this phase, the HR repair pathway is predominant. More specifically, the role of HR to manage the DNA breaks generated by IR in the G2/M phase should be integrated into the RIANS model together with the HR/NHEJ balance that may be dependent on ATM but also on ATM substrates such as MRE11 or BRCA1 [49].

LET is an important factor influencing both cancer induction and radiosensitivity. The RIANS model has been shown to be relevant whatever the LET value. Particularly, the spatial and density distribution of the energy depositions that occur after irradiation is specific to each particle or rays. Notably, the spatial and density distribution of the energy deposition conditions the level of oxidative stress that causes RI ATM monomerization in the cytoplasm and RI DNA breaks in the nucleus. Hence, the ratio between the number of ATM monomers and that of RI DSB directly depends on the LET value and therefore on the corresponding clinical consequences [248]. However, further experiments are needed to verify whether RI cancer risk and radiosensitivity observed clinically may be predicted by in vitro experiments performed with RIANS biomarkers.

The fact that low doses of IR (namely lower than 0.5 Gy) may cause cancer is an important question that has been debated for several decades [249]. The RIANS model has been validated at low doses and provides a biological explanation for hormesis and hypersensitivity to low doses phenomena [250,251]. Furthermore, the RIANS biomarkers have already been validated with two scenarios of CT exams [252,253]. The ratio between the number of ATM monomers and that of RI DSB evoked above with the LET is also an important feature to predict the risk of low doses. For example, while the number of RI DSB is directly proportional to the dose, the number of ATM monomers depends on the amount of X-proteins present in the cytoplasm and therefore on the individual factor. Again, the model of cancer proneness and radiosensitivity proposed in this review must be verified and documented in different irradiation conditions involving low doses to better evaluate the risk of RI cancer and toxicity for each of the genetic syndromes reviewed here.

## 7. Conclusions

This review aimed to survey the major cancer and radiosensitivity syndromes. One of the first conclusions is that the proteins whose mutations are responsible for these syndromes are all phosphorylation substrates of ATM and may present cytoplasmic forms when mutated. The ATM kinase therefore appears at the crossroads of the molecular and cellular bases of cancer proneness and radiosensitivity.

The impairment of the ATM-dependent DNA damage recognition, signaling and repair may cause misrepaired DNA damage that may be either endogenous (e.g., spontaneous DNA breaks) or exogenous (e.g., from the environment). Their formation can be considered as an initiation step in carcinogenesis.

The impairment of the ATM-dependent cell cycle checkpoint control: impaired G2/M and/or G1 arrests may contribute to the propagation of the errors described above. This step can be considered as a promotion step in carcinogenesis.

While these two requirements involve two different functions that should be both impaired, our review shows that the major cancer and radiosensitivity syndromes are caused by one mutation in a single gene. In the frame of the RIANS model, a given protein may ensure its own intrinsic function but may also play additional biological role(s) as a cytoplasmic ATM substrate (called X-protein). Hence, whether spontaneously or after oxidative stress (such as exposure to IR), the flux of ATM monomers from the cytoplasm to the nucleus may be delayed by X-proteins, which may cause impairments of both recognition and repair of DSB via NHEJ and in cell cycle checkpoint control via a limited phosphorylation of CHK1 and/or CHK2 proteins. Such a model appears relevant for the three categories of the major cancer and radiosensitivity syndromes. However, further experiments are needed to better document and consolidate such hypotheses, especially in specific conditions of irradiation.

## Figures and Tables

**Table 1:** Molecular and cellular features of the major syndromes associated with both cancer and individual radiosensitivity.

Cancer Syndrome Category	Gene	Protein	Involvement in DNA Repair	Involvement in Cell Cycle Checkpoint	Syndrome	Type of Cancer	SF2 (%)
Category 1	*ATM*	ATM	X	-	Ataxia Telangiectasia	Leukemia/Lymphoma	1–5
	*ATR*	ATR	X	-	Seckel Syndrome	Leukemia	40–50
	*LIG4*	DNA Ligase 4	X	-	LIG4 syndrome	Lymphoma	2–6
	*XRCC4*	XRCC4	X	-	Microcephalic Primordial Dwarfism	-	nd
	*Art/DCLRE1C*	Artemis	X	-	Artemis Syndrome	Lymphoma	30–50
	*XLF/Cernunnos*	XLF/Cernunnos	X	-	Cernunnos Syndrome	Lymphoma	30–50
	*RAG1* and *RAG2*	RAG1 and RAG2	X	-	Omenn Syndrome	Hepatocarcinoma	30–50
	*NBS*	NBS1	X	-	Nijmegen Syndrome	Lymphoma	5–9
	*MRE11*	MRE11	X	-	Ataxia–Telangiectasia-Like Disorder	Diverse	15–30
	*RAD50*	RAD50	X	-	Nijmegen Breakage Syndrome-Like Disorder	Lymphoma	15
	*XPA* to *XPG*	XP-A to XP-G	X	-	Xeroderma Pigmentosum	Brain and/or skin	15–30
	*BLM/RECQL2*	BLM	X	-	Bloom Syndrome	Sarcoma	15–40
	*WRN/RECQL3*	WRN	X	-	Werner Syndrome	Sarcoma	20–55
	*RTS/RECQL4*	RTS	X	-	Rothmund-Thompson	Sarcoma	30–50
	*MMR* (*hMLH1*, *hMSH2*, *hMSH6*, *hPMS2)*	MMR (MLH1, MSH2, MSH6, PMS2)	X	-	Human Non-Polyposis Hereditary Colon Cancers Syndrome or Lynch Syndrome	Colon, endometrial, ovarian, stomach, small intestine, liver, upper urinary tract, brain, and skin cancers	30–50
	*hMSH2*	MSH2	X	-	Turcot Syndrome	Brain and colon	21–30
	*APC*	APC	X	-	Gardner Syndrome and Turcot Syndrome	Diverse for Gardner and CNS tumors for Turcot	18–30
Category 2	*PI3KCA*	PI3K	-	X	PROS Syndromes	Skin, vasculature, bones, brain	35–40
	*AKT1*	AKT	-	X	Proteus Syndrome	Diverse	nd
	*PTEN*	PTEN	-	X	Cowden Disease	Breast cancer	nd
	*CHK2*	CHK2	-	X	-	Prostate, colon, lung, thyroid, and breast cancer	nd
	*BRCA1*	BRCA1	-	X	Inherited Breast Cancer, Ovarian Cancer	Inherited breast cancer, ovarian cancer	30–50
	*BRCA2*	BRCA2	-	X	Inherited Ovarian Cancer and Male Breast Cancer	Inherited ovarian cancer and male breast cancer	20–40
	*FANC*	FANC	-	X	Fanconi Anemia	Leukemia and breast cancer	15–40
	*RB1*	pRB	-	X	Retinoblastoma Syndrome	Retina cancer	25–35
	*p53*	p53	-	X	Li-Fraumeni	Diverse	20–50
Category 3	*NF1*	NF1 (neurofibromin 1)	-	-	Neurofibromatosis type 1 Syndrome	Benign optic nerve tumor and neurofibrosarcomas, astrocytoma and rhabdomyosarcoma	15–35
	*NF2*	NF2 (schwannomin or merlin protein)	-	-	Neurofibromatosis type 2 Syndrome	Nervous system and schwannomas, meningiomas and ependymomas	12–30
	*TSC1*	Hamartin protein	-	-	TSC Syndrome	Angiofibromas, astrocytomas, renal angiomyolipomas and pulmonary lymphangioleimyomatosis	15–30
	*TSC2*	Tuberin	-	-	TSC Syndrome	Angiofibromas, astrocytomas, renal angiomyolipomas and pulmonary lymphangioleimyomatosis	nd

Nd: not determined.
